# China Registry Study on Cognitive Impairment in the Elderly: Protocol of a Prospective Cohort Study

**DOI:** 10.3389/fnagi.2021.797704

**Published:** 2021-12-23

**Authors:** Yingying Zhu, Dong Pan, Lei He, Xiaoming Rong, Honghong Li, Yi Li, Yaxuan Pi, Yongteng Xu, Yamei Tang

**Affiliations:** ^1^Clinical Research Design Division, Clinical Research Center, Sun Yat-sen Memorial Hospital, Sun Yat-sen University, Guangzhou, China; ^2^Department of Neurology, Sun Yat-sen Memorial Hospital, Sun Yat-sen University, Guangzhou, China; ^3^Guangdong Provincial Key Laboratory of Malignant Tumor Epigenetics and Gene Regulation, Sun Yat-sen Memorial Hospital, Sun Yat-sen University, Guangzhou, China; ^4^Guangdong Province Key Laboratory of Brain Function and Disease, Sun Yat-sen University, Guangzhou, China

**Keywords:** cognitive impairment, dementia, aging, early diagnosis, cohort

## Abstract

**Introduction:** To develop appropriate strategies for early diagnosis and intervention of cognitive impairment, the identification of minimally invasive and cost-effective biomarkers for the early diagnosis of cognitive impairment is crucial and desirable. Therefore, the CHina registry study on cOgnitive imPairment in the Elderly (HOPE) study is designed to investigate the natural course of cognitive decline and explore the clinical, imaging, and biochemical markers for the detection and diagnosis of cognitive impairment on its earliest stage.

**Methods:** Approximately 5,000 Chinese elderly aged more than 50 years were recruited from Sun Yat-sen Memorial Hospital, Sun Yat-sen University in Guangzhou, China by the year 2024. All subjects were invited to complete the clinical assessment, neuropsychological assessment, the biological samples collection (blood and cerebrospinal fluid (CSF)], magnetic resonance imaging (MRI) examination, and optional amyloid and tau PET. The follow-up survey was conducted every 1 year to repeat these assessments for 20 years. To better clarify the relationship between potential risk factors and endpoint events [changes in cognitive score or incidence of mild cognitive impairment (MCI) and/or dementia], appropriate statistical methods were used to analyze the data, including but not limited to, such as linear mixed-effect model, competing risk model, or the least absolute shrinkage and selection operator model.

**Significance:** The CHina registry study on cOgnitive imPairment in the Elderly study is designed to explore the longitudinal changes in characteristics of participants with cognitive decline and to identify potential plasma and imaging biomarkers with cost-benefit and scalability advantages. The results will enable broader clinical access and efficient population screening and then improve the development of treatment and the quality of life for cognitive impairment at the early stage.

**Trial registration number:** NCT04360200.

## Introduction

### Background

With the population aging and life expectancy increasing, dementia has been a major and increasing health challenge for policy makers, healthcare professionals, and family members worldwide (Patterson, 2018; GBD Dementia Collaborators, 2019). The greatest disease burden of dementia has been found in China as approximately 25% of the entire population with dementia live in this middle-income country (GBD Dementia Collaborators, 2019; Jia et al., 2020). Dementia-associated disability and care burden in China will be $1.89 trillion in 2050 (Jia et al., 2018). Therefore, as an important part of public health system of China, it is imperative to focus on the early diagnosis and treatment of cognitive impairment.

Alzheimer’s disease (AD) accounts for 60–80% of total dementia, which maybe most effectively prevented and treated at the earliest and mildest stages (Scheltens et al., 2016). Over past decades, many clinical trials of the anti-AD drugs [e.g., dimebon, (Doody et al., 2008) bapineuzumab, (Salloway et al., 2009) solanezumab, (Doody et al., 2014) bapineuzumab, (Salloway et al., 2014), and verubecestat (Egan et al., 2019)] have failed to prevent, delay, and treat AD. In June of 2021, Food and Drug Administration (FAD) has approved the first AD-treatment drug, aducanumab, which can delay the progression of AD by clearing the amyloid-β (Aβ), (Sevigny et al., 2016; Schneider, 2020) the earliest pathological signature of AD in the brain (Hardy and Selkoe, 2002; Masters et al., 2015; Jack et al., 2018). It further emphasizes the importance of the diagnosis and treatment of cognitive impairment, especially in its early stage.

Currently, cerebrospinal fluid (CSF) testing or PET imaging examination are the only two validated methods to identify Ai deposition in the brain. In consideration of invasiveness or expensiveness, these biomarkers are unsuitable for extensively early screening. Therefore, a minimally invasive, cost-effective marker for the early detection and diagnosis of cognitive impairment is desirable. In recent years, more attention has been paid to screen the valuable biomarkers in peripheral blood (Nabers et al., 2018). Some studies reported that plasma Aβ and phosphorylated-Tau (p-Tau) have high performance in predicting Aβ burden and tau in the brain and CSF, respectively (Nakamura et al., 2018; Janelidze et al., 2020; Karikari et al., 2020). Magnetic resonance imaging (MRI), as a non-invasive and inexpensive technology, also plays an important part in monitoring the AD process. Researchers have found that the structural findings, such as hippocampus volume (Wirth et al., 2013; Toledo et al., 2014), and subcortical changes, such as the manifestations of cerebral small vessel disease (CSVD), (Akoudad et al., 2016; Benjamin et al., 2018; Wang et al., 2020) in MRI may be promising independent markers to predict cognitive impairment. However, less evidence has been found in China. It remains elusive that whether the biomarkers in peripheral blood and parameters in MRI separately or combined can add the predictive value in the early diagnosis of cognitive impairment in the Chinese elderly. Meanwhile, the relation of many risk factors, such as the apolipoprotein E (ApoE) genotype, and lifestyle factors for predicting progression from mild cognitive impairment (MCI) to dementia has been reported in many cohort studies (Cooper et al., 2015; Li et al., 2016). However, these cohorts were conducted in participants with MCI, and the corresponding relation of these factors with the risk of cognitive decline from normal to MCI has not been verified.

Therefore, an ongoing long-term cohort study is conducted in the Sun Yat-sen Memorial Hospital of Sun Yat-sen University, one subcenter of the National Clinical Research Center for Geriatric Disorders, that aims to identify clinical, neuroimaging, and biochemical biomarkers for the early detection and diagnosis of cognitive impairment. The design of the CHina registry study on cOgnitive imPairment in the Elderly (HOPE) study is described in detail in this protocol.

### Objectives

To provide the evidence for the early diagnosis and the treatment of cognitive impairment, this study includes the following objectives:

(1) To investigate the changes in cognitive function and the incidence of MCI and dementia.
(2) To identify the potential markers (clinical, biomedical, and imaging) affecting/predicting the development process of cognitive impairment.
(3) To develop the related prediction models to identify the elderly who are likely to develop MCI and dementia.
(4) To construct a useful database to explore the secular trend of cognitive function in the elderly and to provide a better understanding of the life-course factors having an effect on the aging process.

## Methods

### Study Design

The CHina registry study on cOgnitive imPairment in the Elderly study is designed as a prospective cohort in which the Chinese elderly are followed for up to 20 years. Serial neuropsychological tests, comprehensive biomedical, and imaging examinations are utilized every 1 year to record the changing pattern of cognitive function in the hospital-based Chinese elderly. This protocol is reported according to the Standard Protocol Items: Recommendations for Interventional Trials (SPIRIT; Chan et al., 2013).

### Study Participants and Sample Size

To investigate the natural course of age-related cognitive decline and explore the potential markers for earlier diagnosis of cognitive impairment, participants in the department who are aged 50 years or older and can give informed consent were consecutively recruited from the Department of Neurology, Sun Yat-sen Memorial Hospital (SYSMH), Sun Yat-sen University in Guangzhou, China. The exclusion criteria are as follows: (1) diagnosis with tumors, severe anemia, brain injury, or AIDS); (2) history of psychosis, such as schizophrenia and epilepsy; (3) inability to complete the study protocol, such as physical disability and aphasia; and (4) presence of contraindications for MRI.

The enrollment was started on April 11, 2020 and will be ended on December 31, 2024. The annual number of participants in the Department of Neurology of SYSMH is about 8,000. Therefore, we expect to initially enroll 5,000 population from SYSMH over a period of 5 years. According to the incidence of MCI and dementia (approximately 16 and 6%, respectively) in the Chinese elderly (≥60 years old) (Jia et al., 2020; Lu et al., 2021), this number will be sufficient to conduct longitudinal research on developmental cognitive decline. This study is primarily an epidemiological investigation without any prespecified statistical hypothesis to test, making a prior sample size calculation unnecessary. Enough but not too many elderly participants were enrolled in this project to give our results adequate power in an efficient way. Fortunately, 1,269 participants with a mean age of 66 (±9) years old and 53.4% men have been recruited until October 31, 2021 in this HOPE study.

### Study Procedure

**FIGURE 1:**
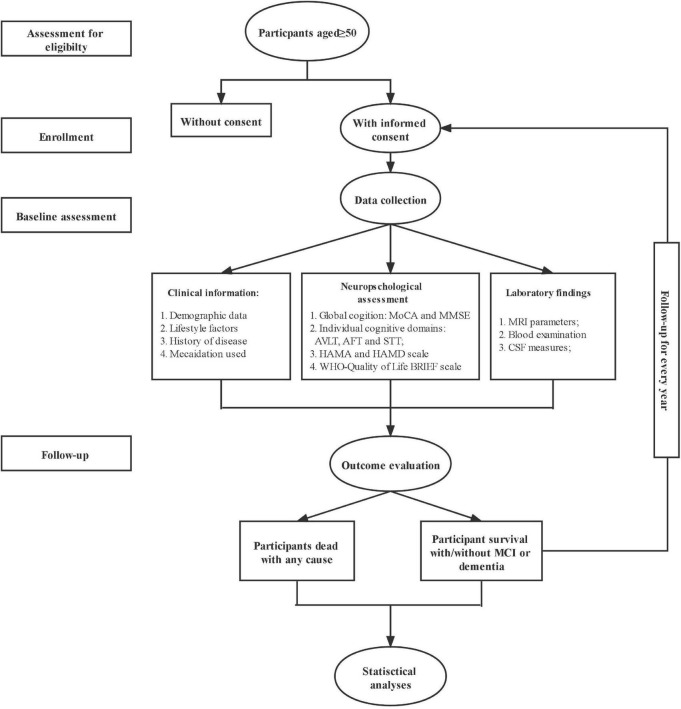
The flow chart of the CHina registry study on cOgnitive imPairment in the Elderly (HOPE) study.

The procedure of the HOPE study is shown in Figure 1. After signing the informed consent form, all eligible subjects with informed consent were invited to complete the baseline survey, such as clinical assessment, neuropsychological assessment, the biological samples collection, and imaging examination. The follow-up survey will be conducted every 1 year for 20 years to repeat the assessments and collect the information of outcomes interested. The schedule of all data collection is shown in Table 1.

**TABLE 1:** The complete overview of data collection and its related tools and time points in the CHina registry study on cOgnitive imPairment in the Elderly (HOPE) study.

Data collected	Measurement tools	Time points	
		Baseline	Each follow-up
Demographic data	Questionnaire	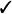	
Lifestyle factors	Questionnaire	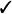	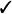
History of disease	Questionnaire and medical records	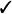	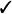
History of medication	Questionnaire and medical records	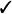	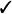
Biomedical biomarkers	Blood sample, CSF sample	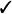	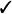
Imaging parameters	MRI, PET	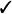	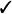
Sleep quality	PSQI; REM-RBD	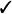	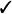
Daily living ability	IADL/ADL	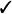	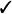
Cognition assessment	MoCA, MMSE, AVLT, AFT, STT,	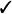	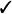
Anxiety	Hamilton anxiety scale	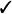	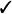
Depression	Hamilton depression scale	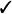	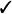
Quality of life	WHO-quality of LifeBREIF	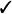	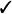

*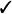 = time of point collecting the corresponding data.*

*CSF, cerebrospinal fluid; MRI, magnetic resonance imaging; PET, positron emission tomography; PSQI, Pittsburgh sleep quality scale; REM-RBD, rapid eye movement sleep behavior disorder screening questionnaire; IADL/ADL, instrumental-/activities of daily living scale; MoCA, Montreal cognitive assessment; MMSE, mini-mental State Examination; AVLT, auditory verbal learning test; AFT, animal fluency test; STT, shape trail test.*

### Outcomes

During the follow-up period, the diagnosis of MCI and AD is based on the core clinical criteria recommended by the National Institute on Aging and the Alzheimer’s Association (NIA-AA) workgroup (Albert et al., 2011; McKhann et al., 2011). Other types of dementia, such as vascular dementia (VaD), Lowy body dementia (LBD), frontotemporal dementia (FTD), and Parkinson’s disease with dementia (PDD), were defined according to globally accepted criteria. The senior clinicians who are in charge of the participants used the criteria to make a diagnosis of MCI and dementia. A seminar was held every week to discuss the uncertainty in diagnosis.

Other than the cognition-related outcomes, the following kinds of diseases were also diagnosed according to the International Classification of Diseases (ICD)-10 and recorded during the study period: all-cause and specific death, cancers, cardiovascular disease (e.g., angina, myocardial infarction, coronary heart disease, and atrial fibrillation) and cerebrovascular disease [e.g., ischemic and hemorrhagic stroke, transient ischemic attack (TIA), and subarachnoid hemorrhage (SAH)].

### Demographic and Clinical Information

A semi-structured questionnaire is used to collect the sociodemographic characteristics of participants (e.g., age, sex, income, education, etc.), habitual dietary intake, and lifestyle factors (e.g., physical activity, smoking status, and alcohol consumption). Daily life ability was examined by the Instrumental-/Activities of Daily Living (IADL/ADL) scale. The Pittsburgh sleep quality index (PSQI) and Rapid Eye Movement Sleep Behavior Disorder Screening Questionnaire (REM-RBD) were used for sleep quality assessment.

The clinical information extracted from the semi-structured questionnaire and medical records includes medical history (e.g., hypertension and diabetes), a medication used (e.g., antidiabetic, blood-lowering, or antiplatelet drugs), routine blood and urine tests (e.g., biochemical tests, thyroid function tests, and urine protein), and physical examination (e.g., weight, height, and blood pressure).

### Neuropsychological Assessments

Tests for the neuropsychological assessments are selected to measure different cognitive domains and global cognition for tracking decline. The neuropsychological battery for different cognitive domains includes episodic memory (Auditory Verbal Learning Test, AVLT), language (Animal Fluency Test), and speed/executive function (Shape Trail Test, STT). The global cognition was tested by Montreal Cognitive Assessment (MoCA)-Beijing version and mini-mental State Examination (MMSE). In addition, the Hamilton Anxiety and Depression (HAMA and HAMD) Scale and WHO-Quality of Life BRIEF scale were implemented to evaluate the neuropsychiatric status and the quality of life.

These neuropsychological assessments were conducted at baseline and each follow-up time by the research staff who is trained and get the qualification certificates.

### Biological Sample Tests

Biological samples (blood and CSF) were collected from those participants who consent at baseline and each follow-up. Ten milliliters of blood collected in the anticoagulant tube were centrifuged at 2,000r/min for 10 min within 8 h. Then, three aliquots of the plasma with 2–3 ml in each polypropylene tube were stored at −80°C. ApoE genotype, Aβ peptide, and p-Tau levels were tested in blood samples. ApoE was genotyped with the method of standardized Sanger sequencing. The commercially available ELISA kit was used to assess the Aβ and p-Tau levels. The rest of the blood plasma was stored for future proteomics and biomarkers testing.

Five milliliters of CSF samples were collected for patients in a left lateral position according to international guidelines (Engelborghs et al., 2017; Shaw et al., 2018). After lumbar puncture, the subjects were supervised for at least 12 h to record any signs of discomfort. CSF collected from each subject was stored at −80°C with 1 ml in each polypropylene tube. The levels of Aβ42/Aβ40 and p-Tau in CSF were tested by using the corresponding ELISA kits.

### Imaging Examination and Analysis

Magnetic resonance imaging was performed by using a 32-channel head coil on 1.5 T or 3.0 T Siemens/Philips Magnetom Trio Tim scanner at the SYSMH for all participants at baseline and each follow-up. The standardized neuroimaging protocol includes three-dimensional T1-weighted imaging (T1WI), T2-weighted imaging (T2WI), magnetic resonance angiography (MRA), diffusion-weighted imaging (DWI), and susceptibility-weighted image (SWI) sequence. The details of the MRI pulse sequence are provided in Table 2. Hippocampal volumes, the manifestation of CSVDs (lacunes, white matter hyperintensitivities (WMHs), and cerebral microbleeds (CMBs), etc.), vascular morphology, and lacunar infarction were obtained according to the corresponding MRI sequence and criteria by two radiologists independently.

**TABLE 2:** The details of the magnetic resonance imaging (MRI) pulse sequence.

MRI unit	Simens 1.5T					Simens 3.0T				
Sequence	T1WI	T2WI	MRA	DWI	SWI	T1WI	T2WI	MRA	DWI	SWI
TR (ms)	1,090	4,500	24	4,000	49	2,000	4,500	21	4,040	27
TE (ms)	8.4	118	7.15	102	40	9	96	3.43	64	20
Flip angle	150	150	23	90	15	150	150	18	90	15
FOV (mm)	230 × 208	230 × 208	230 × 186	230 × 230	230 × 230	220 × 220	220 × 198	200 × 160	220 × 220	220 × 198
Voxel size (mm)	0.4 × 0.4 × 5	0.2 × 0.2 × 5	0.6 × 0.6 × 0.6	1.2 × 1.2 × 5	0.4 × 0.4 × 2.0	0.7 × 0.7 × 5	0.2 × 0.2 × 5	0.3 × 0.3 × 0.6	1.4 × 1.4 × 5	0.9 × 0.9 × 1.5
Slice thickness (mm)	5	5	0.6	5	2	5	5	0.6	5	1.5
Overlapping gap (mm)	1	1	0	1	0	1	1	0	1	0
Acquisition time (min)	2′22′′	2′58′′	5′32′′	42′′	3′53′′	1′22′′	1′39′′	4′28′′	1′30′′	4′54′′

*MRI, magnetic resonance imaging; T1-weighted imaging (T1WI), T2-weighted imaging (T2WI), magnetic resonance angiography (MRA), diffusion-weighted imaging (DWI), and susceptibility-weighted image (SWI); TR, repetition time; TE, echo time; FOV, the field of view; VIBE, volume interpolated body examination; FS, fat-suppression.*

All participants who are suspected with dementia and consented were invited for Aβ PET ([18F] florbetapir (AV-45)) or tau PET ([18F] AV1451or [18F] PI-2620) with the use of PET-CT/MR imaging in 3-dimensional acquisition mode. The brain Aβ and tau quantification were quantified by standardized uptake value ratio (SUVR) value using a composite of the prefrontal, orbitofrontal, parietal, temporal, anterior cingulate, and posterior cingulate and precuneus cortices.

### Quality Control and Queue Maintenance

The completeness and reliability of the questionnaire were checked by the quality control team with the members from the project workforce. For low-quality questionnaires, the participants were further interviewed by another face-to-face or *via* a telephone re-interview rightly to revise the questionnaire.

The data were deposited at an online platform called Research Electronic Data Capture (REDCap). For quality assurance, entry errors were prevented by using a logic check function in the system. The quality control team also checked the data regularly according to the documented questionnaires from the archives to check for quality.

Subjects were interviewed every 1 year for 20 years. If one person misses a visit, the investigator will contact them several times. To reduce the loss follow-up rate, we set an official WeChat account and group for the Hope study to contact participants frequently by posting important notices or reminders for participants, or giving related support and counsel for healthcare in time.

### Statistical Analysis

Results of the HOPE study were interpreted and reported according to relevant guidelines [e.g., STROBE, (Vandenbroucke et al., 2007) TRIPOD (Collins et al., 2015)]. Baseline characteristics of all participants are described. The collected data were presented as mean ± SD or percentages, respectively, and potential group differences were calculated by *t*-test (continuous data) or χ2 (categorical data) or non-parametric test.

Among all participants, the progression rate of normal cognition to MCI, or MCI to dementia, and the incidence rate of MCI, or dementia, or other disease happened during the follow-up period were calculated and put into analyses as binary variables. The changes in the scores, such as cognition, depression, or sleep quality assessments, were recognized with continuous measures in the analyses.

The associations between possible protective or risk factors, endpoint events, and the corresponding prediction models were clarified by using appropriate statistical methods to analyze the data, e.g., logistic regression model (univariate and multivariate), survival curve, or cox regression model for binary variables with time; competing risk model for taking death or other cardio-cerebrovascular diseases into account as competing events with MCI or dementia; linear mixed-effect model for repeated measurement data; and the least absolute shrinkage and selection operator (LASSO) model for developing the predictive model with a large number of predictors. The performance of the developed predictive model were evaluated with discrimination, calibration, and clinical usefulness in the internal validation or even, external validation (if possible). Additional analyses, such as subgroup or stratified analyses, according to any potential confounder (age, sex, history of disease at baseline, etc.) were also conducted. To analyze different trajectories of predictor variables across multiple waves of data, path analyses, and structural equation, modeling were used. Novel statistical methods (e.g., machine learning) may also be tested for multifactorial prediction. Statistical significances were defined as *p* < 0.05 (two-sided).

### Participant and Public Involvement

Neither participants nor the public has been or will be involved in the process of designing, conducting, or reporting this study.

## Ethics and Dissemination

The HOPE study protocol has been approved by the Medical Ethics Committee of Sun Yat-sen Memorial Hospital, Sun Yat-sen University, Guangzhou, China (ID: SYSEC-KY-KS-2020-050). The study protocol has been registered in Clinical Trial (NCT04360200), and any changes to the protocol will be updated on the registry as necessary. Human tissue were used legally in accordance with the Human Biomedical Research Act.

The researcher fully explained the aims and procedure of the HOPE study to all the participants to get their written informed consent. Participants were allowed to withdraw from the HOPE study at any time.

Results from the HOPE study were disseminated by publications in the scientific journals, presentations at both national and international conferences. General results were also shared with participants through mass media releases or public education activities.

## Discussion

This study evaluates the longitudinal progression process of cognitive decline and its related factors within 20 years in the Chinese elderly. The ability of the blood-based biomarkers and the imaging-based parameters in the prediction of cognitive impairment were also explored. Meanwhile, the risk predictive models were developed based on the combination of potential predictors to detect and diagnose cognitive impairment at the early stage.

The prevalence and related disease burden of neuropsychological disease have been increasing worldwide, especially in China (Jia et al., 2018; Zhang et al., 2019). Although the technological advances in CSF biomarkers (Aβ or tau) and imaging examinations (Aβ/tau PET) and increased attention paid to the care of these participants, to some extent, have improved the diagnosis of dementia and increased their quality of life, there is no effective treatment or drugs in those who already have the clinical symptoms of cognitive impairment. FAD recommends that aducanumab should be given only to participants with mild symptoms of AD. Thus, the early detection and diagnosis of cognitive impairment are crucial for the prevention and treatment of dementia.

Treatments for dementia have been recognized to be most effective at the earliest and mildest stages (Sperling et al., 2014; Jack et al., 2018). To facilitate the treatment, supportive biomarker information is necessary to identify the people with high risk at the early stage. Due to high cost and invasion, the PET imaging examination and CSF testing cannot be used on a large scale in the diagnosis of AD. The minimally invasive and cost-effective biomarkers in peripheral blood have been paid attention. Some studies have reported that plasma Aβ40/Aβ42 had high accuracy in predicting brain amyloid-β burden determined by Aβ-PET imaging (Area under the curve (AUC) >90%) (Nakamura et al., 2018). Besides, plasma P-tau181 also had a relation with CSF P-tau181 and the imaging parameter in tau PET (AUC > 85%) (Schindler et al., 2019). However, no evidence of these relations has been found in the Chinese elderly. Furthermore, it remains to be verified whether these plasma biomarkers can predict the risk of MCI and dementia in the Chinese aging population.

Many cross-sectional and few retrospective cohort studies have reported the correlation between imaging markers (lacunes, WMHs, and CMBs), brain atrophy, and the decreased cortical thickness of the cortex in specific regions related to cognition decline, indicating their potential roles in early diagnosis of cognitive impairment (Tekin and Cummings, 2002; Thong et al., 2013; Chen et al., 2015; Habes et al., 2018). However, the results of studies focusing on the relation of imaging parameters and cognition decline are inconsistent (Smith et al., 2000; Jacobs et al., 2012; Ding et al., 2017; Cannistraro et al., 2019; Lombardi et al., 2020). Thus, the possible links between the presence, progression, number, and location of imaging markers and cognitive function still need to be elaborated with high-quality evidence in prospective studies. Besides, whether these imaging parameters can add more power in the prediction of cognitive impairment in the Chinese elderly also needs to be discussed.

Even though the positive associations between many risk factors, such as women, diabetes, depression, and the risk of progression from MCI to dementia, have been verified in many cohort studies (Artero et al., 2008; Richard et al., 2013; Li et al., 2016), whether these risk factors still have an effect on the cognition decline from normal to MCI remains unclear. Thus, the relation or predictive value of these factors in the diagnosis of cognitive impairment on the earliest stage should also be investigated.

Therefore, the HOPE study is conducted to explore the dynamic changes of characteristics of participants with cognitive decline and to identify potential plasma and imaging biomarkers and construct predictive models based on the combinations of predictive factors to achieve early detection and diagnosis of cognitive impairment. In addition, the longitudinal patterns, especially the temporal sequences, of clinical, blood, and imaging markers, which can help us to understand the process of disease progression, are revealed through this project. Therefore, compared with current techniques, the identified diagnostic and prognostic plasma biomarkers and imaging parameters will have the advantages of cost-effectiveness and scalability, enabling broader clinical access and efficient population screening. This will provide evidence to improve the development of treatment and the quality of life for cognitive impairment at the key early stage. Hopefully, the HOPE study can contribute to better recognition of prevention and intervention for dementia, and achievement of optimal dementia healthcare.

## Ethics Statement

The studies involving human participants were reviewed and approved by the medical Ethics Committee of Sun Yat-sen Memorial Hospital, Sun Yat-sen University, Guangzhou, China. The patients/participants provided their written informed consent to participate in this study. Written informed consent was obtained from the individual(s) for the publication of any potentially identifiable images or data included in this article.

## Author Contributions

YT conceived and designed the study. YZ and DP drafted the manuscript. All authors participated in the preparation of this manuscript, played a role in the development of the protocol, and approved the submission of this manuscript.

## Conflict of Interest

The authors declare that the research was conducted in the absence of any commercial or financial relationships that could be construed as a potential conflict of interest.

## Publisher’s Note

All claims expressed in this article are solely those of the authors and do not necessarily represent those of their affiliated organizations, or those of the publisher, the editors and the reviewers. Any product that may be evaluated in this article, or claim that may be made by its manufacturer, is not guaranteed or endorsed by the publisher.

## References

[B1] Akoudad S. Wolters F. J. Viswanathan A. De Bruijn R. F. Van Der Lugt A. Hofman A. (2016). Association of cerebral microbleeds with cognitive decline and dementia. *JAMA Neurol.* 73 934–943. 10.1001/jamaneurol.2016.1017 27271785 PMC5966721

[B2] Albert M. S. Dekosky S. T. Dickson D. Dubois B. Feldman H. H. Fox N. C. (2011). The diagnosis of mild cognitive impairment due to Alzheimer’s disease: recommendations from the national institute on aging-Alzheimer’s association workgroups on diagnostic guidelines for Alzheimer’s disease. *Alzheimers Dement.* 7 270–279. 10.1016/j.jalz.2011.03.008 21514249 PMC3312027

[B3] Artero S. Ancelin M. L. Portet F. Dupuy A. Berr C. Dartigues J. F. (2008). Risk profiles for mild cognitive impairment and progression to dementia are gender specific. *J. Neurol. Neurosurg. Psychiatry* 79 979–984. 10.1136/jnnp.2007.136903 18450788

[B4] Benjamin P. Trippier S. Lawrence A. J. Lambert C. Zeestraten E. Williams O. A. (2018). Lacunar infarcts, but not perivascular spaces, are predictors of cognitive decline in cerebral small-vessel disease. *Stroke* 49 586–593. 10.1161/strokeaha.117.017526 29438074 PMC5832012

[B5] Cannistraro R. J. Badi M. Eidelman B. H. Dickson D. W. Middlebrooks E. H. Meschia J. F. (2019). CNS small vessel disease: a clinical review. *Neurology* 92 1146–1156. 10.1212/WNL.0000000000007654 31142635 PMC6598791

[B6] Chan A. W. Tetzlaff J. M. Altman D. G. Laupacis A. Gøtzsche P. C. Krle C. (2013). SPIRIT 2013 statement: defining standard protocol items for clinical trials. *Ann. Intern. Med.* 158 200–207. 10.7326/0003-4819-158-3-201302050-00583 23295957 PMC5114123

[B7] Chen Y. Wang A. Tang J. Wei D. Li P. Chen K. (2015). Association of white matter integrity and cognitive functions in patients with subcortical silent lacunar infarcts. *Stroke* 46 1123–1126. 10.1161/STROKEAHA.115.008998 25737316

[B8] Collins G. S. Reitsma J. B. Altman D. G. Moons K. G. (2015). Transparent reporting of a multivariable prediction model for individual prognosis or diagnosis (TRIPOD): the TRIPOD statement. *BMJ* 350:g7594. 10.1136/bmj.g7594 25569120

[B9] Cooper C. Sommerlad A. Lyketsos C. G. Livingston G. (2015). Modifiable predictors of dementia in mild cognitive impairment: a systematic review and meta-analysis. *Am. J. Psychiatry* 172 323–334. 10.1176/appi.ajp.2014.14070878 25698435

[B10] Ding J. Sigurðsson S. Jónsson P. V. Eiriksdottir G. Meirelles O. Kjartansson O. (2017). Space and location of cerebral microbleeds, cognitive decline, and dementia in the community. *Neurology* 88 2089–2097. 10.1212/WNL.0000000000003983 28468844 PMC5447401

[B11] Doody R. S. Gavrilova S. I. Sano M. Thomas R. G. Aisen P. S. Bachurin S. O. (2008). Effect of dimebon on cognition, activities of daily living, behaviour, and global function in patients with mild-to-moderate Alzheimer’s disease: a randomised, double-blind, placebo-controlled study. *Lancet* 372 207–215. 10.1016/S0140-6736(08)61074-0 18640457

[B12] Doody R. S. Thomas R. G. Farlow M. Iwatsubo T. Vellas B. Joffe S. (2014). Phase 3 trials of solanezumab for mild-to-moderate Alzheimer’s disease. *N. Engl. J. Med.* 370 311–321. 10.1056/NEJMoa1312889 24450890

[B13] Egan M. F. Kost J. Voss T. Mukai Y. Aisen P. S. Cummings J. L. (2019). Randomized trial of verubecestat for prodromal Alzheimer’s disease. *N. Engl. J. Med.* 380 1408–1420. 10.1056/NEJMoa1812840 30970186 PMC6776078

[B14] Engelborghs S. Niemantsverdriet E. Struyfs H. Blennow K. Brouns R. Comabella M. (2017). Consensus guidelines for lumbar puncture in patients with neurological diseases. *Alzheimers Dement. (Amst)* 8 111–126. 28603768 10.1016/j.dadm.2017.04.007 PMC5454085

[B15] GBD Dementia Collaborators (2019). Global, regional, and national burden of Alzheimer’s disease and other dementias, 1990-2016: a systematic analysis for the Global Burden of Disease study 2016. *Lancet Neurol.* 18 88–106. 10.1016/S1474-4422(18)30403-4 30497964 PMC6291454

[B16] Habes M. Erus G. Toledo J. B. Bryan N. Janowitz D. Doshi J. (2018). Regional tract-specific white matter hyperintensities are associated with patterns to aging-related brain atrophy via vascular risk factors, but also independently. *Alzheimers Dement. (Amst)* 10 278–284. 10.1016/j.dadm.2018.02.002 29644327 PMC5889709

[B17] Hardy J. Selkoe D. J. (2002). The amyloid hypothesis of Alzheimer’s disease: progress and problems on the road to therapeutics. *Science* 297 353–356. 10.1126/science.1072994 12130773

[B18] Jack C. R. Jr. Bennett D. A. Blennow K. Carrillo M. C. Dunn B. Haeberlein S. B. (2018). NIA-AA research framework: toward a biological definition of Alzheimer’s disease. *Alzheimers Dement.* 14 535–562. 10.1016/j.jalz.2018.02.018 29653606 PMC5958625

[B19] Jacobs H. I. Visser P. J. Van Boxtel M. P. Frisoni G. B. Tsolaki M. Papapostolou P. (2012). Association between white matter hyperintensities and executive decline in mild cognitive impairment is network dependent. *Neurobiol. Aging* 33 201.e201–208. 10.1016/j.neurobiolaging.2010.07.015 20739101

[B20] Janelidze S. Mattsson N. Palmqvist S. Smith R. Beach T. G. Serrano G. E. (2020). Plasma P-tau181 in Alzheimer’s disease: relationship to other biomarkers, differential diagnosis, neuropathology and longitudinal progression to Alzheimer’s dementia. *Nat. Med.* 26 379–386. 10.1038/s41591-020-0755-1 32123385

[B21] Jia J. Wei C. Chen S. Li F. Tang Y. Qin W. (2018). The cost of Alzheimer’s disease in China and re-estimation of costs worldwide. *Alzheimers Dement.* 14 483–491. 10.1016/j.jalz.2017.12.006 29433981

[B22] Jia L. Quan M. Fu Y. Zhao T. Li Y. Wei C. (2020). Dementia in China: epidemiology, clinical management, and research advances. *Lancet Neurol.* 19 81–92. 10.1016/S1474-4422(19)30290-X 31494009

[B23] Karikari T. K. Pascoal T. A. Ashton N. J. Janelidze S. Benedet A. L. Rodriguez J. L. (2020). Blood phosphorylated tau 181 as a biomarker for Alzheimer’s disease: a diagnostic performance and prediction modelling study using data from four prospective cohorts. *Lancet Neurol.* 19 422–433. 10.1016/S1474-4422(20)30071-5 32333900

[B24] Li J. Q. Tan L. Wang H. F. Tan M. S. Tan L. Xu W. (2016). Risk factors for predicting progression from mild cognitive impairment to Alzheimer’s disease: a systematic review and meta-analysis of cohort studies. *J. Neurol. Neurosurg. Psychiatry* 87 476–484. 10.1136/jnnp-2014-310095 26001840

[B25] Lombardi G. Crescioli G. Cavedo E. Lucenteforte E. Casazza G. Bellatorre A. G. (2020). Structural magnetic resonance imaging for the early diagnosis of dementia due to Alzheimer’s disease in people with mild cognitive impairment. *Cochrane Database Syst. Rev.* 3:Cd009628. 10.1002/14651858.CD009628.pub2 32119112 PMC7059964

[B26] Lu Y. Liu C. Yu D. Fawkes S. Ma J. Zhang M. (2021). Prevalence of mild cognitive impairment in community-dwelling Chinese populations aged over 55 years: a meta-analysis and systematic review. *BMC Geriatr.* 21:10. 10.1186/s12877-020-01948-3 33407219 PMC7789349

[B27] Masters C. L. Bateman R. Blennow K. Rowe C. C. Sperling R. A. Cummings J. L. (2015). Alzheimer’s disease. *Nat. Rev. Dis. Primers* 1:15056. 10.1038/nrdp.2015.56 27188934

[B28] McKhann G. M. Knopman D. S. Chertkow H. Hyman B. T. Jack C. R. Jr. Kawas C. H. (2011). The diagnosis of dementia due to Alzheimer’s disease: recommendations from the national institute on aging-Alzheimer’s association workgroups on diagnostic guidelines for Alzheimer’s disease. *Alzheimers Dement.* 7 263–269. 10.1016/j.jalz.2011.03.005 21514250 PMC3312024

[B29] Nabers A. Perna L. Lange J. Mons U. Schartner J. Güldenhaupt J. (2018). Amyloid blood biomarker detects Alzheimer’s disease. *EMBO Mol. Med.* 10:e8763. 10.15252/emmm.201708763 29626112 PMC5938617

[B30] Nakamura A. Kaneko N. Villemagne V. L. Kato T. Doecke J. Dor V. (2018). High performance plasma amyloid-β biomarkers for Alzheimer’s disease. *Nature* 554 249–254. 29420472 10.1038/nature25456

[B31] Patterson C. (2018). *World Alzheimer Report 2018.* London: Alzheimer’s Disease International.

[B32] Richard E. Reitz C. Honig L. H. Schupf N. Tang M. X. Manly J. J. (2013). Late-life depression, mild cognitive impairment, and dementia. *JAMA Neurol.* 70 374–382. 10.1001/jamaneurol.2013.603 PMC3694613 23599941

[B33] Salloway S. Sperling R. Fox N. C. Blennow K. Klunk W. Raskind M. (2014). Two phase 3 trials of bapineuzumab in mild-to-moderate Alzheimer’s disease. *N. Engl. J. Med.* 370 322–333. 24450891 10.1056/NEJMoa1304839 PMC4159618

[B34] Salloway S. Sperling R. Gilman S. Fox N. C. Blennow K. Raskind M. (2009). A phase 2 multiple ascending dose trial of bapineuzumab in mild to moderate Alzheimer disease. *Neurology* 73 2061–2070. 10.1212/wnl.0b013e3181c67808 19923550 PMC2790221

[B35] Scheltens P. Blennow K. Breteler M. M. De Strooper B. Frisoni G. B. Salloway S. (2016). Alzheimer’s disease. *Lancet* 388 505–517. 26921134 10.1016/S0140-6736(15)01124-1

[B36] Schindler S. E. Bollinger J. G. Ovod V. Mawuenyega K. G. Li Y. Gordon B. A. (2019). High-precision plasma β-amyloid 42/40 predicts current and future brain amyloidosis. *Neurology* 93 e1647–e1659. 10.1212/WNL.0000000000008081 31371569 PMC6946467

[B37] Schneider L. (2020). A resurrection of aducanumab for Alzheimer’s disease. *Lancet Neurol.* 19 111–112. 10.1016/S1474-4422(19)30480-6 31978357

[B38] Sevigny J. Chiao P. Bussière T. Weinreb P. H. Williams L. Maier M. (2016). The antibody aducanumab reduces Aβ plaques in Alzheimer’s disease. *Nature* 537 50–56. 27582220 10.1038/nature19323

[B39] Shaw L. M. Arias J. Blennow K. Galasko D. Molinuevo J. L. Salloway S. (2018). Appropriate use criteria for lumbar puncture and cerebrospinal fluid testing in the diagnosis of Alzheimer’s disease. *Alzheimers Dement.* 14 1505–1521. 10.1016/j.jalz.2018.07.220 30316776 PMC10013957

[B40] Smith C. D. Snowdon D. A. Wang H. Markesbery W. R. (2000). White matter volumes and periventricular white matter hyperintensities in aging and dementia. *Neurology* 54 838–842. 10.1212/wnl.54.4.838 10690973

[B41] Sperling R. Mormino E. Johnson K. (2014). The evolution of preclinical Alzheimer’s disease: implications for prevention trials. *Neuron* 84 608–622. 10.1016/j.neuron.2014.10.038 25442939 PMC4285623

[B42] Tekin S. Cummings J. L. (2002). Frontal-subcortical neuronal circuits and clinical neuropsychiatry: an update. *J. Psychosom. Res.* 53 647–654. 10.1016/s0022-3999(02)00428-2 12169339

[B43] Thong J. Y. Hilal S. Wang Y. Soon H. W. Dong Y. Collinson S. L. (2013). Association of silent lacunar infarct with brain atrophy and cognitive impairment. *J. Neurol. Neurosurg. Psychiatry* 84 1219–1225. 10.1136/jnnp-2013-305310 23933740

[B44] Toledo J. B. Weiner M. W. Wolk D. A. Da X. Chen K. Arnold S. E. (2014). Neuronal injury biomarkers and prognosis in ADNI subjects with normal cognition. *Acta Neuropathol. Commun.* 2:26. 10.1186/2051-5960-2-26 24602322 PMC4008258

[B45] Vandenbroucke J. P. Von Elm E. Altman D. G. Gøtzsche P. C. Mulrow C. D. Pocock S. J. (2007). Strengthening the reporting of observational studies in epidemiology (STROBE): explanation and elaboration. *PLoS Med.* 4:e297. 10.1371/journal.pmed.0040297 17941715 PMC2020496

[B46] Wang Y. L. Chen W. Cai W. J. Hu H. Xu W. Wang Z. T. (2020). Associations of white matter hyperintensities with cognitive decline: a longitudinal study. *J. Alzheimers Dis.* 73 759–768. 10.3233/JAD-191005 31839612

[B47] Wirth M. Villeneuve S. Haase C. M. Madison C. M. Oh H. Landau S. M. (2013). Associations between Alzheimer disease biomarkers, neurodegeneration, and cognition in cognitively normal older people. *JAMA Neurol.* 70 1512–1519. 10.1001/jamaneurol.2013.4013 24166579 PMC4962545

[B48] Zhang Y. Guan Y. Shi Z. Yue W. Liu S. Liu S. (2019). Sex differences in the prevalence of and risk factors for cognitive impairment no dementia among the elderly in a rural area of Northern China: a population-based cross-sectional study. *Neuroepidemiology* 52 25–31. 30476921 10.1159/000493141

